# Role of Dietary Carbohydrates in Cognitive Function: A Review

**DOI:** 10.1002/fsn3.70516

**Published:** 2025-07-01

**Authors:** Muhammad Tayyab Arshad, Sammra Maqsood, Rawan Altalhi, Ghalia Shamlan, Isam A. Mohamed Ahmed, Ali Ikram, Muhammed Adem Abdullahi

**Affiliations:** ^1^ Functional Food and Nutrition Program, Faculty of Agro‐Industry Prince of Songkla University Songkhla Thailand; ^2^ University Institute of Food Science and Technology The University of Lahore Lahore Pakistan; ^3^ National Institute of Food Science and Technology University of Agriculture Faisalabad Faislabad Pakistan; ^4^ Department of Biological Sciences, College of Science University of Jeddah Jeddah Saudi Arabia; ^5^ Department of Food Sciences and Nutrition, College of Food and Agricultural Sciences King Saud University Riyadh Saudi Arabia; ^6^ Department of Food Science and Postharvest Technology, Jimma University College of Agriculture and Veterinary Medicine Jimma University Jimma Ethiopia

**Keywords:** carbs, diet, malnutrition, mental health

## Abstract

Dietary carbohydrates play an important role in mood regulation, mental health, and cognitive performance, which is driving rapid development in studies on the diet‐brain interaction. The review will focus on the intricate relationship between brain function and carbohydrate intake, emphasizing how different types of carbohydrates impact mental health and cognitive outcomes. Simple carbohydrate intake (often known as “sugars”) is consistently linked to a decline in overall cognition, while complex carbohydrate intake is linked to both short‐ and long‐term memory improvement and successful brain aging. Dietary macronutrients affect the brain and cognition through a variety of pathways, including glucose and insulin metabolism, neurotransmitter effects, and cerebral oxidation and inflammation. Carbohydrate supplements appear to enhance cognitive function in people who engage in prolonged, high‐intensity physical activity with high energy requirements.

## Graphical abstract

The review will focus on the intricate relationship between brain function and carbohydrate intake, emphasizing how different types of carbohydrates impact mental health and cognitive outcomes. We look at how carbohydrates impact brain energy metabolism, specifically how the brain utilizes glucose as the primary fuel source and how consuming too little carbohydrate affects emotional stability and cognitive functioning.

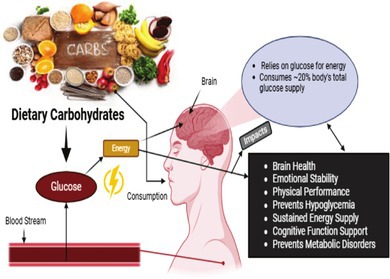

## 1 Introduction

It is increasingly recognized that human health involves understanding the interaction between diet, brain function, and psychological well‐being. Proper nutrition is vital for the proper development, upkeep, and functioning of the brain, thus directly manipulating mood and thought procedures. These functions may be cooperated by nutritional insufficiencies or disparities, which can subsidize to mood disorders and cognitive deterioration (Gardener and Rainey‐Smith 2018).

Energy and nutrients are significant for brain functioning; hence, consuming a well‐adjusted diet full of energetic nutrients is significant. Metabolic paths of energy are vital to both nutrition and neuroscience (Gan et al. 2024). The brain consumes 20% of the body's energy in spite of only weighing 2% of its physique (Melzer et al. 2021). It is important to consume macronutrients such as proteins, fats, and carbohydrates to meet this energy need for optimal brain function (Chu et al. 2022). Carbohydrates are the body's key fuel for the brain subsequently glucose metabolism withstands thinking procedures such as memory, consideration, and judgment making (Ekstrand et al. 2021). Overall, diet had an effect on neurogenesis and neuroplasticity, two processes that are essential for learning and adaptability (Monti et al. 2015).

Nutrition also has a drastic impact on the biochemical environment of the brain, which subsequently influences one's mental well‐being. For instance, as Moore et al. (2018) described, micronutrients such as B vitamins and omega‐3 fatty acids are intricate in neurotransmitter amalgamation accountable for leading reactivity to anxiety and mood. Nutritional interferences, predominantly those concentrating on nutrient rich mealtimes, play a critical function in the consequence of mental health, demonstrated by concentrated depression and nervousness scores, as investigations have revealed (Banjari et al. 2014).

People whose diets are high in processed foods and complex carbohydrates are more likely to suffer from psychological distress and cognitive damage (Rahman et al. 2022). There is a link among the gut–brain axis and psychological and emotional health with concern to food, as recent investigation has revealed (de Sousa Rodrigues et al. 2017). Food affects brain role through immunological and neuroendocrine mechanisms that permit through the gut microbiota. Goyal et al. (2015) recognized that this binary way association regulates how our brainpowers grow. Fiber and prebiotic ironic diets, for instance, improve the variety of gut microbiota, which improves mental role and stress pliability (Wahl et al. 2016).

Adequate nutrition is necessary for the cognitive function and mental well‐being. Diets containing whole grains, fruits, vegetables, and healthy fats have been associated with neurodegenerative diseases (Liang et al. 2023). Diets such as the Mediterranean diet and the MIND diet have been proven to slow down cognitive impairment (Berendsen et al. 2018). These food patterns contain antioxidants and anti‐inflammatory substances, which protect against neuroinflammation and oxidative stress, two of the most significant factors associated with cognitive impairments due to aging (Table 1) (Cheng et al. 2024; Allès et al. 2012).

**TABLE 1:** Dietary carbohydrates, sources, and implications for brain health.

Type	Source	Role	References
Simple carbohydrates	Sugars (e.g., glucose, fructose in fruits, honey)	Immediate energy for brain function may cause rapid glucose fluctuations, impacting mood and cognitive clarity	(Benton 2018; Amjad et al. 2021; Dienel 2019)
Complex carbohydrates	Whole grains, legumes, and vegetables	Sustained energy release due to slower digestion, supporting stable glucose levels crucial for cognitive performance and memory	(Korczak et al. 2016; Melzer et al. 2021; Monti et al. 2015)
Dietary fiber	Vegetables, fruits, whole grains, and nuts	It supports gut microbiota, enhances gut–brain communication, and reduces neuroinflammation linked to cognitive decline	(Goyal et al. 2015; Wahl et al. 2016; Ekstrand et al. 2021)
Glycogen	Synthesized in the body from carbohydrates consumed	Acts as an emergency glucose reserve for brain function during fasting or intensive cognitive demands	(Magistretti and Allaman 2022; Falkowska et al. 2015)
Resistant starch	Green bananas, cooked and cooled rice/potatoes	Fermented by gut microbiota to produce short‐chain fatty acids, potentially improving neuroprotective effects and reducing inflammation	(Zhang, Li, et al. 2024; Clemente‐Suárez et al. 2022; Hanhineva et al. 2010)
Low‐glycemic carbohydrates	Lentils, oats, nonstarchy vegetables	Promote steady glucose availability, reducing oxidative stress and enhancing sustained attention and memory	(Ekstrand et al. 2021; Amjad et al. 2021)
High‐glycemic carbohydrates	White bread, sugary cereals, processed foods	Rapid glucose release can cause spikes and crashes, potentially impairing concentration and increasing neuroinflammation	(Wahl et al. 2016; Melzer et al. 2021)
Ketogenic‐compatible carbohydrates	Berries, low‐carbohydrate vegetables	Limited carbohydrate intake in ketogenic diets shifts energy metabolism to ketones, which may support brain energy efficiency and protect against cognitive decline	(Rahman et al. 2022; Moore et al. 2018)
Fructooligosaccharides (FOS)	Onions, garlic, asparagus, leeks	Act as prebiotics to support gut health, indirectly enhancing brain function via the microbiota–gut–brain axis	(Goyal et al. 2015; Wahl et al. 2018; McKenna et al. 2012)
Sugar alcohols	Sorbitol, xylitol, erythritol	Provide a low‐calorie sweetener alternative with minimal impact on blood glucose, though excessive consumption may affect gut health and cognition	(Chen et al. 2023; Banjari et al. 2014; Melzer et al. 2021)
Lactose	Dairy products	It provides glucose and galactose, which is important for neuronal energy metabolism, though lactose intolerance may reduce its availability for some individuals	(Steiner 2019; Dienel 2017)
Glucose metabolism enhancers	Polyphenols (berries, tea), omega‐3 sources	Enhance glucose uptake and utilization in the brain, promoting better cognitive health and protecting against metabolic dysfunctions	(Monti et al. 2015; Moore et al. 2018; Rahman et al. 2022)
Carbohydrates in combination with protein	Whole grain with nuts, legumes	Combining protein and carbohydrates stabilizes glucose levels and supports neurotransmitter synthesis, such as serotonin (linked to mood regulation)	(Wahl et al. 2016; Melzer et al. 2021; Korczak et al. 2016)
Excessive refined carbohydrates	Sweets, pastries, sugar‐sweetened beverages	Linked to increased risk of insulin resistance and inflammation, contributing to impaired memory and increased risk of neurodegenerative diseases	(Berendsen et al. 2018; Clemente‐Suárez et al. 2022; Dienel 2019)

From academic achievements by children to adults' productivity, nutritional interventions help improve cognitive functioning in almost all scenarios. Carbohydrates serve as a source of energy that is important for the continuation of longer cognitive processes, particularly with mentally stressful activities (Wasyluk et al. 2019).

In addition, diets that have a higher proportion of complex carbohydrates to simple sugars tend to stabilize blood glucose levels, thereby reducing mood swings and cognitive fatigue (Amjad et al. 2021). With the connection between nutrition, brain health, and psychological well‐being comes the requirement to have dieting patterns that help promote a healthy brain throughout life. The better that studies are carried out in assessing how individual nutrients and the diet as a whole will influence one's mind, the strategies for boosting cognitive and emotional well‐being via nutrition will come into play. They are the primary source of energy for neurons and glial cells, carbohydrates are essential to brain function. The brain mainly uses glucose, which is produced through the metabolism of carbohydrates, for energy‐intensive functions such as synaptic activity, neurotransmitter production, and signal transmission (Gardener and Rainey‐Smith 2018). Glucose metabolism is a part of cognitive processes, such as memory, attention, and decision‐making, it is, therefore, important to eat adequate carbohydrates for brain health, according to Melzer et al. (2021).

The human brain consumes approximately 120 g of glucose daily, accounting for almost 20% of the body's total energy consumption. The demand emphasizes the necessity of a stable supply of glucose from dietary carbohydrates (Ekstrand et al. 2021). Whole grains and legumes are complex carbs that gradually release glucose, supporting long‐term cognitive function and reducing fatigue when performing cognitively demanding tasks (Meeusen 2014).

Simple carbohydrates, such as refined sugars, cause rapid elevations and subsequent declines in blood glucose levels, which can impact mood regulation and cognitive function (Monti et al. 2015). Besides its influence on the gut–brain axis, carbohydrates also impact brain health. High‐fiber carbohydrates, like those in fruits, vegetables, and whole grains, promote the growth of healthy gut flora. The short‐chain fatty acids of the microbes modify brain function by decreasing inflammation and enhancing neuroplasticity (Goyal et al. 2015). Regular consumption of carbohydrates has also been linked with enhanced cognition and reduced risk of neurodegenerative disease such as Alzheimer's in older adults (Rahman et al. 2022).

Carriers exhibit a neuroprotective function through maintenance of the equilibrium of the brain as we age, according to recent findings. Cognitive resistance and decreased aging in the brain were noted among models of animals under diets comprising more carbohydrates and minimal protein (Wahl et al. 2016).

This diet resembles improved sensitivity in the direction of insulin, leading to glucose digestion and usage by the brain (Moore et al. 2018). Moreover, the neurotransmitter serotonin, which controls mood, is influenced by carbohydrate intake, which is essential for psychological health. Increased consumption of carbs raises serotonin levels, which in turn increases tryptophan availability (Banjari et al. 2014). Other studies have shown that consuming more carbohydrates reduces depression and anxiety. (Berendsen et al. 2018).

The energy compulsory to perform psychological and emotional roles is delivered by carbs, interpreting them vital for brain role. Modest carb consumption, explicitly of complex carbohydrates, improves psychological well‐being, guards against decrease in cognitive progressions, and subsidizes to long‐term cognitive purpose (Scholey 2023). Further studies into carbohydrate metabolism and its influence on brain function will enhance nutritional guidance for different groups. The objective of this review is to highlight the intricate relationship between the consumption of carbohydrates and psychological well‐being by identifying the ways in which carbohydrates impact energy metabolism, neurotransmitter function, cognitive function, and temperament.

## 2 Role of Carbohydrates in Brain Energy Metabolism

**FIGURE 1:**
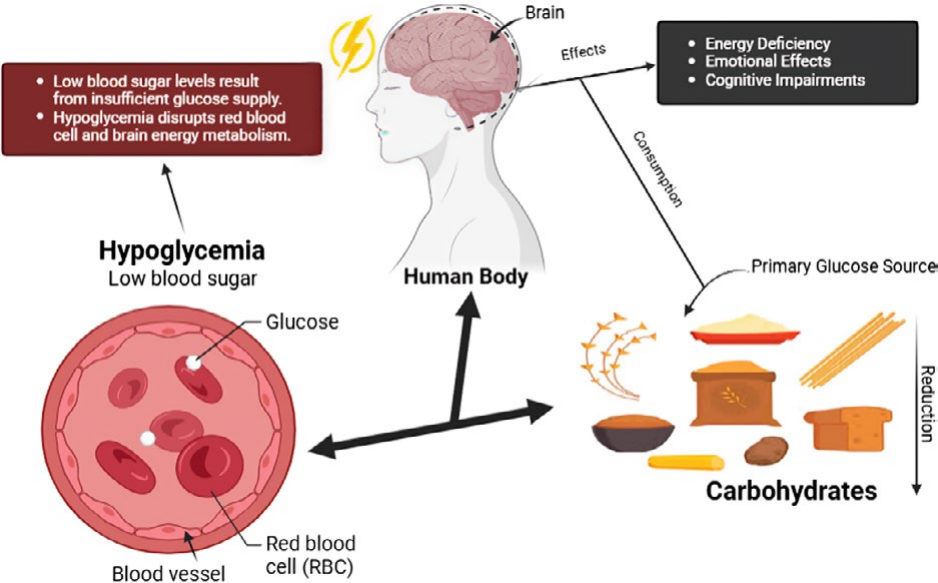
Impact of low carbohydrate intake on hypoglycemia and brain health.

Glucose is the main fuel for the brain, and therefore, carbs are very crucial in metabolic production of energy (Figure 1). The great metabolic demand of brain functions such as synaptic transmission, ion movement, and neurotransmitter cycling is the basis for glucose dependence (McKenna et al. 2012). Brain is special in that it needs a constant and tightly controlled supply of glucose from the bloodstream, eating carbohydrates is necessary in order to maintain cognitive function (Falkowska et al. 2015).

Crucial to glucose metabolism, astrocytes convert glucose to lactate prior to transferring it to neurons for oxidative energy production (Duan et al. 2025). The support of neuronal activity and plasticity relies on this process (Magistretti and Allaman 2022). This mechanism focuses on the astrocyte‐neuron lactate shuttle process, which is part of maintaining mental performance and the assurance of energy efficiency through the coordination of several types of brain cells (Dienel 2019).

Glycogen stored in astrocytes is both a storage facility and an immediate energy source, most notably when the brain is especially active (Falkowska et al. 2015). Low glucose availability can have serious adverse effects on mood and cognitive functions, as in hypoglycemia or carbohydrate deprivation. Hypoglycemia impairs neuronal communication and neurotransmitter synthesis, damaging brain function, and the person experiences decreased sharpness of mind and weariness (Dienel 2019). A steady carbohydrate supply is needed for emotional health, for chronic imbalances in glucose supply have also been linked to mood disorders like anxiety and depression (Aucoin and Bhardwaj 2016).

In neurodegenerative diseases such as Alzheimer's, the relationship between glucose metabolism and brain health is most obvious when glucose utilization in impaired brain areas is often reduced (Zhang et al. 2021). This metabolic dysfunction, often termed “Type 3 diabetes,” underscores the role that carbohydrates play in preserving cognitive function and delaying the onset of neurodegenerative diseases (González et al. 2022). In addition, the brain's versatility in exploiting alternative energy substrates, including ketones, over extended intervals of carbohydrate deprivation demonstrates that it is metabolically versatile but also poses questions for long‐term implications of diets of low carbohydrates (Dienel 2017).

Dietary balance on carbohydrate metabolism also impacts the gut–brain axis because it can influence the gut microbiota, subsequently used to mediate mental health (Chojnacki et al. 2023). In fact, according to Wachsmuth et al. (2022), prebiotics, most of which are made of complex carbohydrates, stimulate good bacteria in the gut that change the function of the brain due to microbial metabolites produced during metabolism, such as short‐chain fatty acids. This complex interaction highlights the wide‐ranging effects of carbohydrate intake on both brain energy metabolism and mental health.

As a result, carbs play a significant role in brain energy metabolism, cognitive function, and emotional stability. The diverse functions of dietary carbs, including the gut–brain axis and the astrocyte‐neuron lactate shuttle, are of major importance for the proper functioning of the brain.

### 2.1 Glucose: Primary Energy Source

**FIGURE 2:**
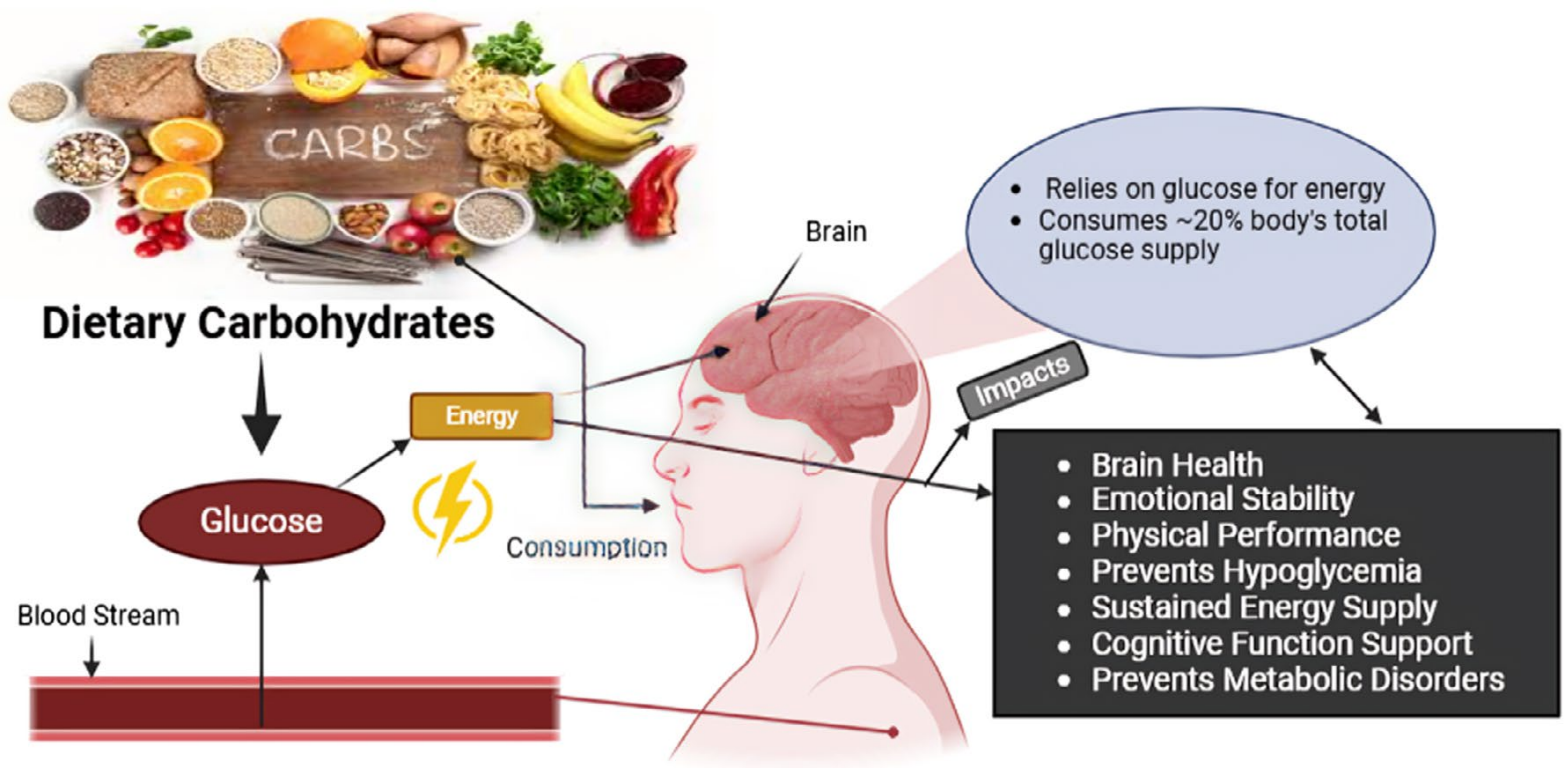
Benefits of adequate carbohydrate intake on brain.

As the brain's primary energy substrate because it is required for the continued maintenance of neuronal activity and integrity of cognitive processes, glucose constitutes approximately 20% of total glucose usage by an adult despite contributing to only 2% of the body's total weight (Figure 2). These high demands can be explained by the ongoing energy needs for ion transport, neurotransmitter production, and synaptic transmission—all of which are essential for healthy brain function (McKenna et al. 2012).

The brain relies on a continuous supply of glucose from the blood because it does not have substantial energy reserves compared to other tissues. This dependence underlines the significance of eating sufficient carbs from foodstuff for the brain to function at its highest (Falkowska et al. 2015). Glucose metabolism in the brain is controlled by astrocytes, which are the main glial cells in the brain. After taking up glucose from the blood, they perform glycolysis to generate lactate, which is then supplied to neurons. A cost‐effective method of energy generation, the astrocyte‐neuron lactate shuttle supports the high metabolic needs of neuronal cells (Magistretti and Allaman 2022).

Glycogen, an astrocytic glucose stand‐in, has appeared as a vigorous storage of energy throughout hyperactivation of the mind and fleeting glucose deficiency (Falkowska et al. 2015). Quick worsening in brain function might take place when glucose source is negotiated, such as in hypoglycemia. Meanwhile optimum functioning of the brain depend on upon steady obtainability of glucose, metabolic turbulences may modify mood, severe fatigue, and cognitive damage (Fioramonti and Pénicaud 2019). There is further evidence that connects neurodegenerative diseases, such as Alzheimer's with sustained alterations in glucose availability, which exacerbate cognitive impairment (Dienel 2019).

The glucose generated through the degradation of ingested polysaccharides and disaccharides is absorbed into the blood and then transported to the brain for the activation of carbohydrate metabolism. Singh et al. (2017) suggested that neurons and astrocytes process glucose glycolysis to produce pyruvate. Pyruvate is then involved in the tricarboxylic acid cycle (TCA) in the mitochondria, in which it gets converted into adenosine triphosphate (ATP). Neuronal communication and synaptic function, which underpin cognition, memory, and learning, are powered by ATP (McKenna et al. 2012).

Glucose metabolism directly provides energy and contributes to the production of neurotransmitters. For example, glutamate and gamma‐aminobutyric acid (GABA), neurotransmitters important for excitatory and inhibitory communication, respectively, are precursors of metabolites of glucose (Dienel 2019).

This metabolic function relates glucose availability to the cognitive and functional capabilities of the brain. The metabolism of carbohydrates is also a factor for oxidative stress management in the brain. When antioxidant mechanisms cannot fully counteract ROS, which is one of the products of glucose oxidation in mitochondria, components of biology can suffer oxidative injury (Zhang, Liu, et al. 2024). Disruptions in metabolic diseases or age‐related decline of glucose metabolism worsen ROS accumulation, leading to neuron damage and worsening loss of cognitive function (Bayliak et al. 2023).

Dietary carbohydrates also regulate the gut‐brain axis, and this influences brain health. Complex carbohydrates increase the richness of gut bacteria and promote the synthesis of short‐chain fatty acids, which regulate energy metabolism and inflammation in the brain. This interaction further underscores the systemic effects of carbohydrate metabolism on mental and emotional health (Wachsmuth et al. 2022).

Impaired glucose metabolism has implications for mood and cognitive performance. Research indicates that stable glucose levels are associated with optimal mental performance, and low‐carb diets or sustained glucose deficiency impair executive function, memory, and attention (Muth and Park 2021). These findings underscore the critical importance of a balanced carbohydrate diet in maintaining cognitive functions across the lifespan. Glucose is necessary for brain energy metabolism to support neuronal signaling, synapse function, and neurotransmitter production. The interaction between astrocytic and neuronal glucose metabolism the function of glycogen as an energy store, and the dietary effect of carbs on the gut–brain axis underscore carbohydrate metabolism's importance to health in the brain. Therefore, a stable glucose supply forms the basis of sustaining cognitive functions and lowering the risk of neurological and neurodegenerative diseases.

## 3 Effects of Hypoglycemia or Low‐Carbohydrate Intake

**FIGURE 3:**
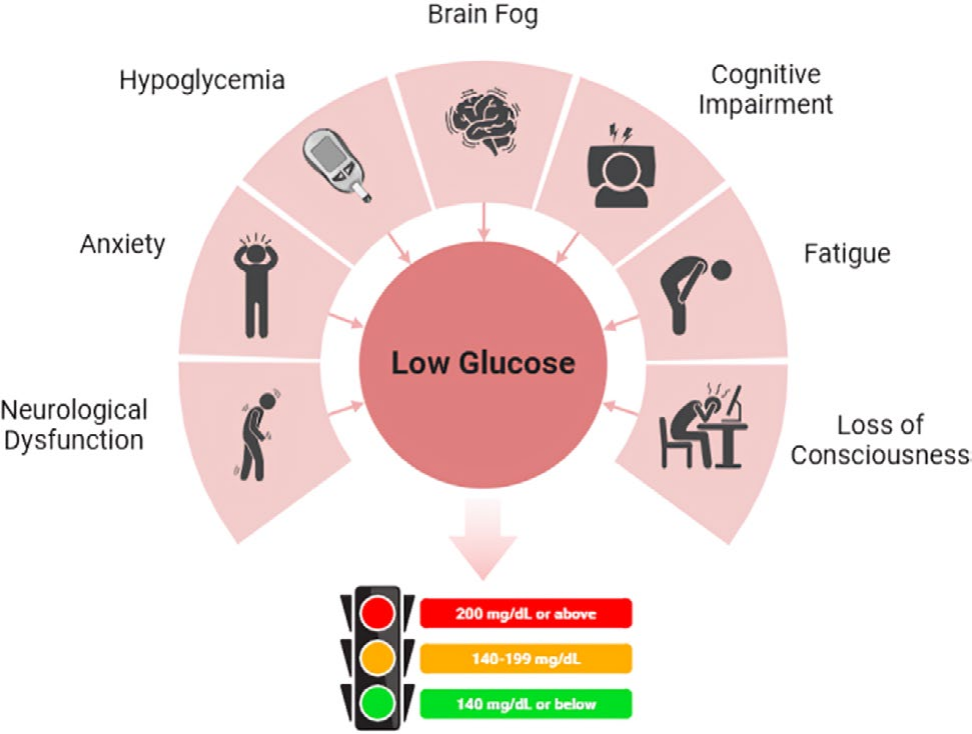
Complications of low glucose level.

Glucose is the brain's primary fuel; hypoglycemia, caused by abnormally low blood glucose levels, significantly affects mood and mental health (McKenna et al. 2012). Low glucose availability impairs cognitive functions, such as memory, attention, and decision‐making skills, which are often associated with mood disorders such as irritation, anxiety, and fatigue (Figure 3) (Falkowska et al. 2015). These effects demonstrate the need for stable blood sugar levels to maintain healthy mental and emotional states. This occurs due to neurocognitive deficits associated with hypoglycemia, where there is a depletion of neuronal energy that, consequently, results in the inhibition of synaptic activity and generation of neurotransmitters, including glutamate and gamma‐aminobutyric acid (GABA) (Dienel 2019). Such deficits have effects that translate to decreased mental acuity and slower processing speed. For example, poor judgment, diminished working memory, and defective executive functioning have been identified as acute hypoglycemia‐related issues.

According to Bayliak et al. (2023), more frequent of low blood sugar might also lead to worsening oxidative stress in the brain, leading to long‐term damage of neurons and cognitive loss. Although they do an excellent job of helping in weight management, low‐carb diets can induce mild hypoglycemia, particularly during the early adaptation phase, impacting emotional and cognitive stability. According to studies, carbohydrate limitations in diets reduce glucose supply, causing mental fatigue and an inability to concentrate. This effect is evident when a person performs demanding cognitive work, whereby glucose availability is critical in producing the best output from the brain (Muth and Park 2021).

The primary cause of hypoglycemia‐linked mood disorders is a disruption in brain energy homeostasis. The main features are restlessness, irritation, and increased stress response caused by the inability of the brain to meet its metabolic needs. This better illustrates the broader impact that glucose metabolism has on psychological well‐being; chronic low‐carbohydrate dieting has also been related to an increased incidence of anxiety and depressive symptoms (Ebrahimpour‐Koujan et al. 2019).

A consistent carbohydrate diet has been shown to enhance temperament and cognitive abilities. Incessant glucose resource positive discrimination that increases brain transmission and neurotransmitter fabrication while improving emotional steadiness and psychological clarity (Zhang et al. 2021). The importance of consuming a diet low in carbohydrates in order to maintain well‐intentioned psychological and passionate well‐being.

### 3.1 Types of Carbohydrates and Their Cognitive Effects

The biochemical configuration and physiological influence of simple and complicated carbohydrates are what fixed them apart in the methods these disturb and reinforce cognitive capabilities. Glucose and sugars, which are binary modest carbohydrates, are quickly broken down and produce energy speedily. Cognitive capabilities such as consideration and memory are frequently improved at pivotal occurrences of great mental process, for instance, through this outpouring of glucose. These are shadowed by a precipitous decrease in blood glucose points, which can reason fatigue and compromised mental developments (Wasyluk et al. 2019).

Beans and whole grains are certain complex carbohydrates that produce glucose gradually, upholding energy levels continuously. Policymaking and problem‐solving roles, which are reliant on a stable supply of glucose, are based on this (Ooi et al. 2011).

A diet rich in carbohydrates has been related to a healthier mood and memory because it delivers the brain with continuous energy (Tay et al. 2016). One of the methods carbs impact blood sugar levels is through the glycemic index (GI). To ensure long‐term intellectual performance and avoid oxidative stress, complex low‐GI carbohydrates should be eaten (Clemente‐Suárez et al. 2022).

High GI foods, often simple carbohydrates, may eventually affect mood and cognitive function because of fluctuating blood sugar levels and inflammation (Amjad et al. 2021). A diet of carbohydrates should be well balanced, with preference given to complex and low‐GI sources. It ensures a constant glucose supply required for neurotransmitter production and neuronal activity required for mental function (Table 2) (Muth and Park 2021).

**TABLE 2:** Types of carbohydrates and their cognitive effects.

Category	Detail	Effects	References
Simple vs. complex carbohydrates	Simple carbohydrates include sugars that provide quick energy but may lead to rapid spikes and drops in blood glucose levels. Complex carbohydrates include starches and fiber, leading to slower digestion and a steady release of glucose	Simple carbohydrates may cause rapid mood fluctuations, whereas complex carbohydrates can enhance sustained energy and focus	(Amjad et al. 2021; Muth and Park 2021; Ooi et al. 2011)
Glycemic response	High‐glycemic foods cause rapid increases in blood sugar, whereas low‐glycemic foods result in gradual glucose absorption	High‐glycemic foods are linked to impaired memory and focus; low‐glycemic foods improve mood stability and attention span	(Breymeyer et al. 2016; Philippou and Constantinou 2014; Grout et al. 2023)
Mood regulation	Glycemic index and glycemic load influence neurotransmitter synthesis and stress responses	A low glycemic load reduces stress and anxiety; a high glycemic load is associated with depression and mood swings	(Rahimlou et al. 2018; Carneiro and Leloup 2020; Guillou et al. 2023)
Fiber‐rich carbohydrates	High fiber intake slows glucose absorption and promotes gut health, indirectly supporting cognitive health	Sustains attention and reduces mental fatigue through stable blood sugar levels	(Ohlsson et al. 2017; Gao et al. 2019; Aziz et al. 2024)
Impact on Children and adolescents	Carbohydrate intake affects memory, attention, and behavior, particularly in school performance	High‐glycemic diets are linked to hyperactivity and attention deficits; balanced intake improves cognitive functioning	(Wasyluk et al. 2019; Drozdowska et al. 2021)
Carbohydrates and sleep	Postexercise meals with low glycemic index improve sleep quality; high glycemic index meals have disruptive effects	Better sleep is associated with improved cognitive performance and mood the next day	(Vlahoyiannis et al. 2018; Benton et al. 2022)
High‐carbohydrate diets in chronic conditions	High‐carbohydrate diets in type 2 diabetes show varying cognitive outcomes depending on glycemic index	Low‐glycemic carbohydrates improve memory and executive function in diabetic individuals	(Liu et al. 2024; Tay et al. 2016; Grout et al. 2023; Bonsembiante et al. 2021)
Interaction with neurotransmitters	Carbohydrate metabolism influences neurotransmitters like serotonin and dopamine	Balanced carbohydrate intake enhances mood and cognitive resilience	(Guo et al. 2021; Dhailappan and Samiappan 2022; Gao et al. 2019)
Ketogenic diet and carbohydrate restriction	Low‐carbohydrate, high‐fat diets reduce glucose reliance and promote ketone usage	Improves attention and executive function in certain populations	(Cohen et al. 2018; Zhang 2023)

### 3.2 Simple vs. Complex Carbohydrates

These show significant differences in their structures, digests, and glycemic response. These differences cause immense alterations in the brain function of subjects. Since simple carbohydrates comprise glucose and fructose‐containing one or two sugar units, they can easily be absorbed and digested quickly with a significant increase in blood glucose concentration.

**FIGURE 4:**
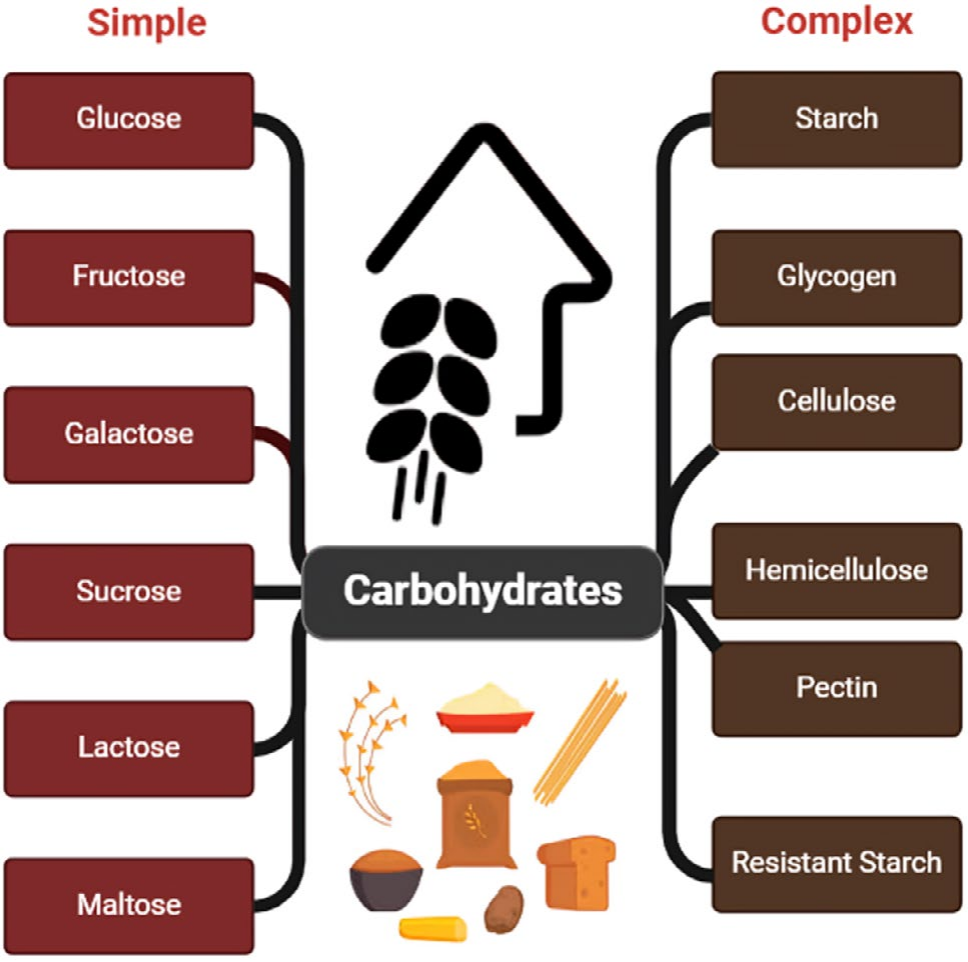
Simple and complex carbs.

This short‐lived increase in energy might, at times, improve memory and attention, but often results in “sugar crashes,” a sharp decline in energy and cognitive function (Wasyluk et al. 2019). For example, whole grains, legumes, and vegetables contain long chains of sugar molecules that constitute complex carbs (Figure 4). Consequently, glucose is gradually introduced into the bloodstream because digestion occurs more slowly (Ooi et al. 2011). These cognitive abilities, including working memory, attention, and decision‐making, are supported long‐term if the energy supply is uniform and ongoing (Tay et al. 2016).

The glycemic index (GI) indicates the rate at which a carbohydrate‐containing food raises blood glucose. Simple carbohydrate foods typically have a high GI, leading to glucose spikes that eventually can lead to inflammation and oxidative stress that can impact cognitive function (Amjad et al. 2021). Low‐GI complex carbs, by contrast, provide for steady glucose delivery that supports neurotransmitter activity and the energy needs of brain cells (Clemente‐Suárez et al. 2022).

Frequent consumption of complex carbohydrates, especially those rich in dietary fiber, is associated with a reduced risk of neurodegenerative diseases and long‐term cognitive benefits (Muth and Park 2021). Although they provide instant energy, simple carbs should be consumed in moderation to avoid adverse effects on brain health, such as mood swings and decreased cognitive flexibility (Breymeyer et al. 2016).

### 3.3 High‐Glycemic vs. Low‐Glycemic Foods

**FIGURE 5:**
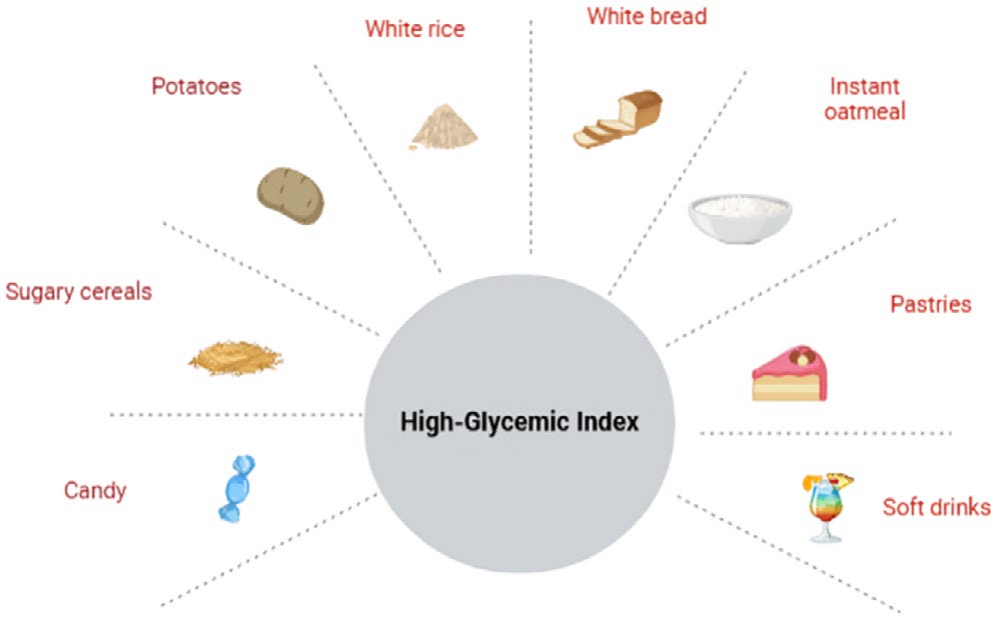
High glycemic index foods.

Food intake significantly affects mood and cognitive function due to the glycemic reaction of foods. HGI foods are examples of foods that cause the blood glucose level to elevate fast and then fall (Figure 5) (Haleem 2023). Hyperglycemia fluctuation, especially during prolonged mental work, has been shown to influence attention, memory, and overall cognitive performance (Philippou and Constantinou 2014).

Because of the sharp decline in glucose availability that occurs after consuming HGI meals, mood disorders such as irritation and exhaustion are particularly common (Haghighatdoost et al. 2016). These foods include whole grains, legumes, and non‐starchy vegetables since their slower release of glucose means steadier levels, which enhance cognitive endurance, according to Drozdowska et al. (2021).

Studies show that a group of people on an LGI diet have greater success on tests that demand the ability to focus over the long term and remember to recall (Grout et al. 2023). Depression and anxieties induced by glycemic instability are less likely to arise when on an LGI diet because swings in blood sugar are minimized (Rahimlou et al. 2018). Glucose is the brain's primary source of fuel, variations in its availability directly affect neurotransmitter production and neuronal transmission. Eating meals with high HGI can create transient euphoria as a result of the rapid access to glucose, but it typically culminates in a crash that decreases mood and mental function (Breymeyer et al. 2016).

On the contrary, low‐glycemic index (LGI) food has been linked for a long time to better mental and emotional well‐being and stress tolerance (Daniel et al. 2019). Haleem (2024) quotes a study that indicates adding low‐GI carbohydrates to regular meals can enhance mood and cognitive function.

This dietary strategy guards against cognitive loss brought on by metabolic stress and promotes sustained mental function (Carneiro and Leloup 2020). Carbohydrate type and glycemic response considerably impact mood, cognitive function, and brain health (Bruta et al. 2021). Complex carbohydrates provide your brain with a steady supply of glucose, which improves long‐term cognitive function and emotional regulation, even though they provide you energy right away. High‐glycemic diets might interfere with mood and compromise cognitive performance due to the fast swings in blood sugar. On the contrary, low‐glycemic diets support steady energy levels and improve mental and emotional health. After some time, a diet that stresses complex, low‐GI carbs can also improve mood problems and cognitive function. Fiber‐rich carbohydrates greatly enhance cognitive performance and combat fatigue by promoting steady energy release and overall metabolic well‐being. Examples of high‐dietary fiber foods include whole grains, legumes, fruits, and vegetables (Wang et al. 2022). These foods have a low GI and slowly release glucose into the bloodstream. This steady energy supply is required for continuous attention and long‐term optimal brain function (Grout et al. 2023).

To diminish mental fatigue, fiber slows down the rate of carbohydrate digestion and leads to a more consistent glucose level, thereby sustaining the sensation of satiety. This helps diminish the glucose fluctuations often associated with high‐GI, low‐fiber diets that cause energy crashes and interfering behaviors (Drozdowska et al. 2021). Clemente‐Suárez et al. (2022) discovered that having stable levels of glucose enhances concentration and executive function by allowing regular brain communication and neurotransmitter production. The gut‐brain axis links the microbiome with brain function, and fiber in food keeps the gut healthy. Studies have demonstrated that short‐chain fatty acids, which are created when the gut digests fiber, can influence cognitive function and reduce inflammatory markers associated with fatigue (Carneiro and Leloup 2020).

Longitudinal research has demonstrated that the consumption of fiber‐rich carbs is able to enhance mood and reduce the risk of fatigue‐related disorders. An example is the interaction between fiber intake and reduced symptoms of fatigue; this is due to the fact that fiber enhances sustained energy levels and reduces metabolic stress due to refined carbohydrates (Amjad et al. 2021). Adding these carbohydrates to your diet will make you mentally alert, concentrate for longer durations, and have healthy cognitive function.

## 4 Carbohydrates and Neurotransmitter Synthesis

Of all the macronutrients, carbohydrates play an important role in the brain's metabolism and cognition. Glucose, a product of carbohydrate metabolism, is the primary fuel for brain cells. Carbohydrates play a role in neurotransmitter production, which enables the brain to communicate due to their impact on glucose metabolism and the gut‐brain axis.

As stated by Arens (2018), fermentation of intestinal fiber alters the gut microbiota and is the first step in the relationship between carbohydrates and neurotransmitter production. Fermentation of the carbohydrate, specifically fiber, in the enormous intestine leads to the founding of short‐chain fatty acids (SCFAs) like acetate, propionate, and butyrate. These SCFAs can moderate neurotransmitter amalgamation and have the capability to irritate the blood brain barricade. Accumulative the obtainability of carbohydrates in the hindgut can upsurge the fabrication of mood regulating neurotransmitters, in specific serotonin, as per investigation lead by Gao et al. (2019).

Ashurova et al. (2023) distinguished that the metabolism of the vital amino acid tryptophan into serotonin by gut microbes disturbs mood, reasoning, and emotional directive. By the interface of nutrition, gut health, and brain activity, this classification founds a connection among the ingestion of carbs in the diet and the amalgamation of important neurotransmitters in the brain (Gao et al. 2019).

Dietary carbohydrates impact serotonin, a neurotransmitter related to cognitive role, mood moderation, and well‐being. Serotonin is a precursor of tryptophan, and its metabolism can be influenced by carbohydrate availability. Carbohydrates improve insulin release, which helps to increase the amount of tryptophan in the blood and the uptake of certain amino acids into the muscle. Tryptophan can then be utilized by the brain to produce serotonin. For optimum serotonin synthesis, mood, and cognitive role, the consumption of an adequate quantity of carbohydrates is essential (Ximenes‐da‐Silva and Guedes 2020).

Gao et al. (2020) established that the ingestion of carbohydrates can have a positive effect on tryptophan metabolism, supporting the premise that dietary carb is vital for the regulation of serotonin and enhanced brain function.

Dopamine, a neurotransmitter related to attention, reward, and motivation, is just one of numerous neurotransmitters that are affected by carbs, including serotonin. In fact, it is indeed true that the high carb diet increases the supply of tryptophan to the brain, but it also influences the production of dopamine and norepinephrine as well, both of the most critical neurotransmitters. These neurotransmitters are used in cognitive functions such as learning, memory, and attention. In their investigation on the effects of a high‐fat and high‐sugar diet on neurotransmitter metabolism, Guo et al. (2021) found that these types of diets alter the composition of the gut microbiota, which further impacts the neurotransmitter systems of the brain. Their work revealed that the consumption of carbohydrates is significant in the maintenance of neurotransmitter levels, which are vital to appropriate brain function.

### 4.1 Influence of Carbohydrate Intake on Mood and Cognitive Function

Carbohydrates are not just accountable for fabricating neurotransmitters but also for the directive of temperament, cognitive capabilities, and brain well‐being overall. Glucose fluctuations, especially those resulting from diets greater in refined carbs or sugars, can damage psychological and emotional functioning. The extended time it takes the body to break down complex carbohydrates such as vegetables and whole grains into glucose that the mind is capable of using as fuel. Low‐glycemic index carbohydrates increase mood and sleep, subsequently improving memory and concentration. The kind of carbohydrate expressively supports the amalgamation of neurotransmitters, according to Benton et al. (2022). Based on Benton et al. (2022), this investigation demonstrated that the ingesting of diverse carb types may contribute to preserving brain function and constraining variations in mood.

Eating carbs in the technique of high fiber foodstuffs is the utmost operative process to supply the brain with a continuous stream of glucose. In accumulation to providing the brain with a continuous source of energy, these nutrients impact the gut bacteria and those stimuli the amalgamation of neurotransmitters (Bordone et al. 2019). A diet high in fiber improves cognitive function by increasing emotional steadiness, decreasing fatigue, and improving concentration, as per investigation.

Additionally, serotonin and other neurotransmitters are prejudiced by the modifications in the microbiota persuaded by a high fiber diet (Nair and Nair 2020). The metabolic function of dietary carbohydrates in neurotransmitter metabolism underscores the reputation of adequate carbohydrate ingestion for psychological well‐being and optimum brain function (Dhailappan and Samiappan 2022).

### 4.2 Mechanisms of Carbohydrates in Modulating Brain Function

Carbohydrates apply a robust effect on brain functioning partially due to the circumstance that these will certify an equilibrium of energy in the mind. Glucose is vital for the functioning of the brain at its optimal, meanwhile it is an energy luxurious organ. Glucose turbulences can subsidize to cognitive damage in the form of thoughtfulness and memory glitches. Ensuing the consequences of Bayliak et al. (2023), it is vital to have a constant source of glucose and, by inference, carbohydrates in order to function properly in the brain, especially as we age.

Bayliak et al. (2023) demonstrated how oxidative stress, changed neurotransmitter production, and cognitive impairment can be caused by disruptions in glucose metabolism. Mood, sleep, and brain activity are all influenced by the circadian rhythm, which is regulated by carbs. Memory recall and enduring attention tests are two places where the timing and composition of carbs affect intellectual function (Muth and Park 2021). Taking carbohydrates at morning or lunchtime raises cognitive capacity and emotional balance and maintains blood sugar in the brain level all day long (Muth and Park 2021).

All things taken into account, carbohydrates are absolutely critical to neurotransmitter synthesis and brain function. They influence the synthesis of serotonin, dopamine, and other neurotransmitters through their effects on glucose metabolism and the gut–brain axis. Fiber‐rich carbohydrates help maintain cognitive function, raise mood, and reduce fatigue by promoting stable glucose levels (Daulay et al. 2022).

The availability of carbohydrates is closely associated with neurotransmitter production, such as serotonin, especially if the gut flora is involved. It appears that carbohydrates are critical nutrients for optimal brain function, hence the best cognitive performance and mental wellness as the research unfolds to help understand the complex relationships between nutrition, neurotransmitter production, and brain function (AlAmmar et al. 2020).

Serotonin is a neurotransmitter that regulates mood, appetite, and sleep, primarily produced from carbohydrates. Carbohydrate consumption increases the production of insulin, which promotes the extraction of other amino acids in the blood and enhances tryptophan uptake in the brain (Gao et al. 2019).

Tryptophan, one of the serotonin precursors, directly correlates with serotonin synthesis. Consequently, diets full of carbs convert tryptophan into serotonin more freely; this could positively impact mood and psychological well‐being (Ximenes‐da‐Silva and Guedes 2020). Serotonin production is the primary mediator of the link between eating carbohydrates and improving mood. This procedure allows the brain to absorb tryptophan, which increases serotonin levels. This procedure has been associated with better mood and emotional health (Muth and Park 2021).

Carbohydrate ingestion has been indicated to be inversely related to irritability and mood disorders, whereas moderate carbohydrate intake might improve mood, possibly through enhanced serotonin activity (Benton et al. 2022). Although serotonin is produced based on carbs, excessive carb intake, especially refined sugars, can contribute to addictive behaviors. High sugar intake can cause high peaks and crashes in blood sugar levels, leading to binge eating and craving cycles (Guo et al. 2021). Because sugary foods make dopamine release, which in turn strengthens the need for more sweet foods, this behavior is also similar to addiction. Besides, studies show that constant consumption of highly processed sugars changes the brain's reward system, which is also akin to the effects of substance use disorders (Avena 2023). These potentially addictive behaviors require awareness of how to balance the danger of overconsumption with the amounts of carbohydrates consumed for enhancing mood (Gearhardt and Schulte 2021).

## 5 Psychological Impacts of Carbohydrate‐Restricted Diets

The psychological effects of low‐carb diets, including the ketogenic diet, on mood, cognition, and emotional regulation, have been studied. It has been reported that these diets may have a dual effect: some report feeling happier and more focused because their blood sugar levels are stable, whereas others report mood swings, irritability, or difficulty concentrating during the adaptation period (Sindler et al. 2024). By stabilizing neuronal activity, the impact of the ketogenic diet on brain metabolism, especially ketone body synthesis, may help individuals feel less anxious and happier (Zheng et al. 2024).

Other research even indicates improvement in cognition, especially in patients suffering from neurological disorders such as epilepsy or Alzheimer's disease (Bellamy 2024). However, individuals who have a history of eating disorders will find emotional stress with these diets due to their restrictive nature (Table 3) (Oliveira et al. 2021).

**TABLE 3:** Psychological impacts of carbohydrate‐restricted diets.

Focus area	Findings	References
Effects on mood	A systematic review found significant reductions in depression and anxiety among individuals following low‐carbohydrate diets, though effects varied by baseline mood disorders	(Varaee et al. 2023; Sindler et al. 2024)
Cognition	Improved focus and cognitive performance were observed during ketogenic diet adherence, attributed to ketone utilization by the brain	(Garner et al. 2024; Mentzelou et al. 2023)
Emotional regulation	Reduction in emotional lability and better stress management reported among ketogenic diet adherents in a clinical trial	(Bellamy 2024; Zheng et al. 2024)
Brain adaptation to ketones	The brain's metabolic flexibility improves with sustained ketone use, enhancing neuroprotection and reducing neuroinflammation	(Shahpasand et al. 2024; Anderson et al. 2024)
Psychological challenges	Participants in a study reported feelings of deprivation and food guilt during the initial stages of low‐carbohydrate diets, particularly among those with disordered eating tendencies	(Oliveira et al. 2021; Hu et al. 2022)
Adherence benefits	Positive reinforcement through weight loss and improved mental clarity motivated participants to maintain low‐carbohydrate diets long term	(Bellamy 2024; Adams et al. 2023)
Neurological implications	Ketogenic diets showed potential in managing symptoms of neuropsychiatric conditions like bipolar disorder and depression, preventing relapse in clinical cases	(Walaszek et al. 2024; Mentzelou et al. 2023)
Appetite and cravings	Improved appetite control and reduced cravings were reported, likely due to stabilized blood sugar and ketone‐related satiety effects	(Zheng et al. 2024; Kusmy et al. 2024)

### 5.1 Ketogenic Diet and Psychological Well‐Being

This can be shown by the improvement of individuals who are suffering from mood disorders like anxiety and depression (Amirinejad et al. 2022). Evidence shows that ketosis has the potential to alter how neurotransmitters function, particularly to create an increase in GABA or gamma‐aminobutyric acid, which calms down the brain (Walaszek et al. 2024).

In general, the evaluation of randomized controlled trials suggested that a low‐carb diet produced clinically relevant improvements in anxiety and depression symptoms, and this was noted for people on this diet. This implies that diet therapies might be added as adjuvants to conventional treatments in mental health (Varaee et al. 2023; Lei 2022).

Additionally, those following a ketogenic diet were often better regulated emotionally, perhaps due to the more stable energy ketones provide to the brain, as ketones are more reliable than glucose in supporting neural activity (Garner et al. 2024).

### 5.2 Cognitive and Emotional Challenges of Carbohydrate Restriction

On the other hand, carbohydrate restriction could cause psychological challenges. Especially during the first days or weeks of initiation, some people can experience “keto flu,” a short‐term but intense mixture of symptoms, such as mood swings, lethargy, and irritation (Mentzelou et al. 2023).

Most researchers believe that the symptoms associated with these individuals are brought on by the change from glucose to ketone bodies used primarily by the brain as fuel. Specifically for individuals with tendencies of mood disorders, withdrawal symptoms caused by carbohydrates may lead to a disturbance in regulating their emotions due to emotional stress and mental exhaustion (Sindler et al. 2024).

Further, long‐term carbohydrate restriction might lead to binge eating and food‐related guilt in certain people, especially those predisposed to disordered eating patterns (Oliveira et al. 2021).

Diets lower in carbohydrates, particularly ketogenic diets, have complex and multidimensional psychological effects. Though these diets may propose benefits such as enhanced temperament regulation, intellectual functioning, and reduced anxiety, they also have damaging psychological effects, particularly throughout the initial stages of dietary adaptation. Achieving the extreme benefits of carbohydrate‐controlled diets while reducing prospective negative effects needs a stable method considering each individual's mental and physiological requirements.

### 5.3 Adaptations of the Brain for Neurological Health

Generally, glucose is the main fuel of the mind. However, during carbohydrate‐restricted periods such as when brought about by ketogenic diets, the body shifts and uses ketones, more specifically beta‐hydroxybutyrate, acetoacetate, and acetone, as the primary fuel source (Daneshzad et al. 2020). This adaptation is imperative for preserving brain functions when the glucose supply is curtailed. Rather than glucose, the brain cells use ketones, which are created when the liver processes fatty acids (Tzenios and Wong 2024).

Due to the fact that it provides the brain with a more stable and effective source of energy, this adaptation is especially beneficial for those on ketogenic diets, as studies have shown that it enhances concentration and cognitive function (Clemente‐Suárez et al. 2022). Neuroprotection and brain plasticity could be two other advantages of ketogenic diets over better energy metabolism. Ketones have neuroprotective effects through the reduction of inflammation and oxidative stress, which are the key drivers of neurodegenerative diseases (Shahpasand et al. 2024).

Scientific research has established that ketosis relieves symptoms of neurodegenerative disorders such as MS, epilepsy, and Parkinson's disease and retards Alzheimer's disease‐associated amyloid plaque progression (Bellamy 2024). This implies that ketogenic diets can potentially be therapeutic in treating or preventing neurodegenerative diseases by reinforcing brain resilience and function. Ketones also alter the expression of some BDNF involved in learning, memory, and synaptic plasticity, states Anderson et al. (2024). This implies that, especially in old age or patients with cognitive decline, ketogenic diets can enhance cognition by increasing the synthesis of BDNF (Mentzelou et al. 2023).

Furthermore, Zheng et al. (2024) states that these neuroplastic changes could also be a factor for improved mental well‐being through the enhanced regulation of mood and overall emotional state.

### 5.4 Potential Implications for Neurological Health

There will be profound impacts on neurological well‐being due to the shift to ketone metabolism. The first is that it can assist the brain in coping with metabolic stress. Cells in neurodegenerative diseases might not respond to glucose any more, but ketosis can supply a different source of energy, potentially arresting the disease (Noakes et al. 2023).

Furthermore, ketogenic diets have shown impressive clinical benefits by lowering seizures in epilepsy, especially among drug‐resistant individuals (Varaee et al. 2023). Although ketosis has promising neuroprotective benefits, it is essential to remember that not all individuals can take advantage of these diets. Especially at the beginning of the adaptation phase of the diet, some may experience adverse effects such as mood swings or cognitive fogginess. However, once the body adapts to the metabolism of ketones, most of the symptoms are eradicated (Sindler et al. 2024).

## 6 Psychological Challenges and Benefits of Adhering to Carbohydrate‐Restricted Dietary Patterns

Even more are aware of the psychological benefits behind maintaining low‐carb diets, like a ketogenic diet, which has enhanced mood and overall emotional well‐being. Studies showed that the regulation of neurotransmitters‐serotonin and GABA, which would control mood and stress, was modified by the ketosis metabolic state, as described by Walaszek et al. (2024).

Low‐carb diets help stabilize the mood by lowering insulin swings and maintaining blood sugar levels, which might reduce the symptoms of depression and anxiety (Garner et al. 2024). It has been found in studies that people on low‐carb diets, particularly those suffering from mood disorders, experienced significant reductions in their symptoms of anxiety and depression (Varaee et al. 2023). In addition, ketogenic diets have been linked to enhanced cognitive function, which can positively impact psychological well‐being. Improved concentration, alertness, and mental acuity may occur due to the brain's ability to utilize ketones as an alternative fuel source (Bellamy 2024).

Most individuals discover that they become more satiated as a result of these cognitive gains on low‐carbohydrate diets. Moreover, these benefits can lead to greater efficiency, daily functioning, and satisfaction with life overall. A low‐carbohydrate diet does have some good impacts on mental health but also has some challenges. For people with an eating disorder or binge history, stress and guilt about food can be a significant challenge (Beard et al. 2022).

Lowered carb consumption is linked with greater food cravings and thoughts, and in turn can increase shame and resentment in individuals who have had previous histories of eating disorders (Oliveira et al. 2021). Such emotions may be a source of trouble in upholding a diet regimen and may consequently result in periods of constraint and binge eating. These may be tremendously interesting to ride out the “keto flu,” or early version of a ketogenic diet.

As renowned by Sindler et al. (2024), when the body is producing the transition from sweltering glucose to ketone, this may manifest with fatigue, petulance, headaches, and temper swings. An ephemeral decrease in mental health could be instigated by the intense influence of these indications on mood and emotional regulator (Zheng et al. 2024). For persons who are even now fighting mental sicknesses such as anxiety or desperateness, these momentary moods might aggravate them.

### 6.1 Long‐Term Adherence and Psychological Sustainability

This may be difficult to uphold a low‐carb diet for the longer period of time. Most individuals struggle to maintain a very low‐carb diet based on cultural food habits, social restrictions, and the temptation of easily accessible carbohydrate‐based foods. Additionally, individuals are prone to growing higher levels of stress and emotional misery due to isolation and deprivation triggered by dietary restriction (Clemente‐Suárez et al. 2022).

Psychological resilience of these diets must be tested since the limitation over extended periods may trigger weariness or abnormal eating behavior. On the contrary, those whose carbohydrate‐restricted diets are maintained succeed in showing improved control of their eating behaviors, improvements in body image, and a sense of achievement, factors that improve psychological well‐being, according to Garner et al. (2024). Of course, the diet also feels sustainable and even rewarding because the mental benefits of the treatment include improvements in mood and cognitive performance, as well as a reduction in anxiety, as observed by Bellamy (2024).

Following a carbohydrate‐restricted diet, especially ketogenic ones, can elicit several positive psychological responses, including improved mood, cognitive performance, and emotional control. These benefits must be weighed, however, against the psychological disadvantages of dieting‐cum‐cravings, feelings of guilt over food, and the painful early stages of adjustment to dieting. Ketogenic diets may, in themselves, have the potential to influence mental and neurological function positively; however, each body responds differently to the diet, and not everyone may be able to maintain that diet over long periods. A good approach to the development of individual dietary routines that optimize the benefits conferred by carbohydrate restriction while keeping off the dangers associated with restrictive eating behaviors requires psychological effects, positive and otherwise.

## 7 Brain–Carbohydrate Relationship

The human brain requires carbohydrates as essential nutrients to perform all its functions throughout its life cycle. Under normal conditions, the brain uses glucose, which is derived from carbs, as its primary source of energy. However, during periods of metabolic adaptation, other energy sources, such as ketones, may also be important (Goyal et al. 2017).

Optimization of cognitive health across life must be understood by relating the intake of carbohydrates and brain function at different phases of life, such as in childhood, maturity, and older adults (Table 4) (Del Moro et al. 2022). This paper examines how carbohydrate consumption relates to brain development, cognition, and aging across significant phases of life. In addition, it considers the effect of starvation and poor dietary practices on neurological health.

**TABLE 4:** Brain carbohydrates relationship across the life stage.

Stage	Key insights	Findings	References
Childhood	Carbohydrates play a crucial role in cognitive development and learning. Early nutritional deficiencies can lead to long‐term cognitive impairments	Digestible carbohydrates are essential for energy metabolism in infants and toddlers, promoting learning and memory development Malnutrition in rats during early stages is linked to impaired brain development	(Stephen et al. 2012; Alamy and Bengelloun 2012; Langley‐Evans 2015; Ilich and Brownbill 2010)
Adulthood	Moderate carbohydrate intake supports cognitive performance, energy regulation, and stress resilience. High glycemic diets may impair attention and memory	Low glycemic index diets improve cognitive performance in adults during tasks requiring sustained attention Balanced carbohydrate intake supports brain health across adulthood	(Meeusen 2014; Kim et al. 2024; Schurr 2014; Sünram‐Lea and Owen 2017; Langley‐Evans 2015)
Aging	Carbohydrates are critical for mitigating cognitive decline. Adequate glucose supports brain metabolism, while low glucose levels may exacerbate dementia risk	Decline in brain aerobic glycolysis with aging impacts memory and cognitive flexibility Mediterranean diets, rich in complex carbs, are linked to a reduced risk of dementia in older adults	(Goyal et al. 2017; Knight et al. 2016; González‐Reyes et al. 2016; Zhu et al. 2015; Schurr 2014; Le Couteur et al. 2016)
Across the lifespan	Early‐life nutrition influences long‐term brain health. Lifespan studies suggest a need for balanced carbohydrate intake to optimize neurodevelopment and protect against age‐related diseases	Early‐life nutritional programming impacts longevity and cognitive health The life‐cycle approach highlights the interconnectedness of nutrition during critical developmental stages for brain health	(Vaiserman 2014; Parida 2024; Rong et al. 2018; Bernstein and McMahon 2022; Markussen et al. 2024; Kim et al. 2024; Milosavljevic et al. 2022)

### 7.1 Carbohydrates and Brain Development in Childhood

Childhood is a period of rapidly developing and growing the human brain, which highly necessitates energy. Carbohydrates, in terms of glucose, fuel the growing brain in an event to increase its energy. Bernstein and McMahon (2022) found that high formation rates of new connections in the brain make the early years of life vulnerable to dietary effects. Because glucose availability during this critical time promotes processes including synaptic plasticity, neurotransmitter production, and myelination, all of which are required for cognitive and motor skills glucose availability is crucial for neurodevelopment (Stephen et al. 2012).

Lack of adequate early‐life carbohydrate intake, for instance, caused by malnutrition, has been associated with later effects on brain function. For instance, studies based on animal models have established that restricted carbohydrate availability could affect synaptic plasticity and cognitive function at critical brain development periods (Alamy and Bengelloun 2012).

**FIGURE 6:**
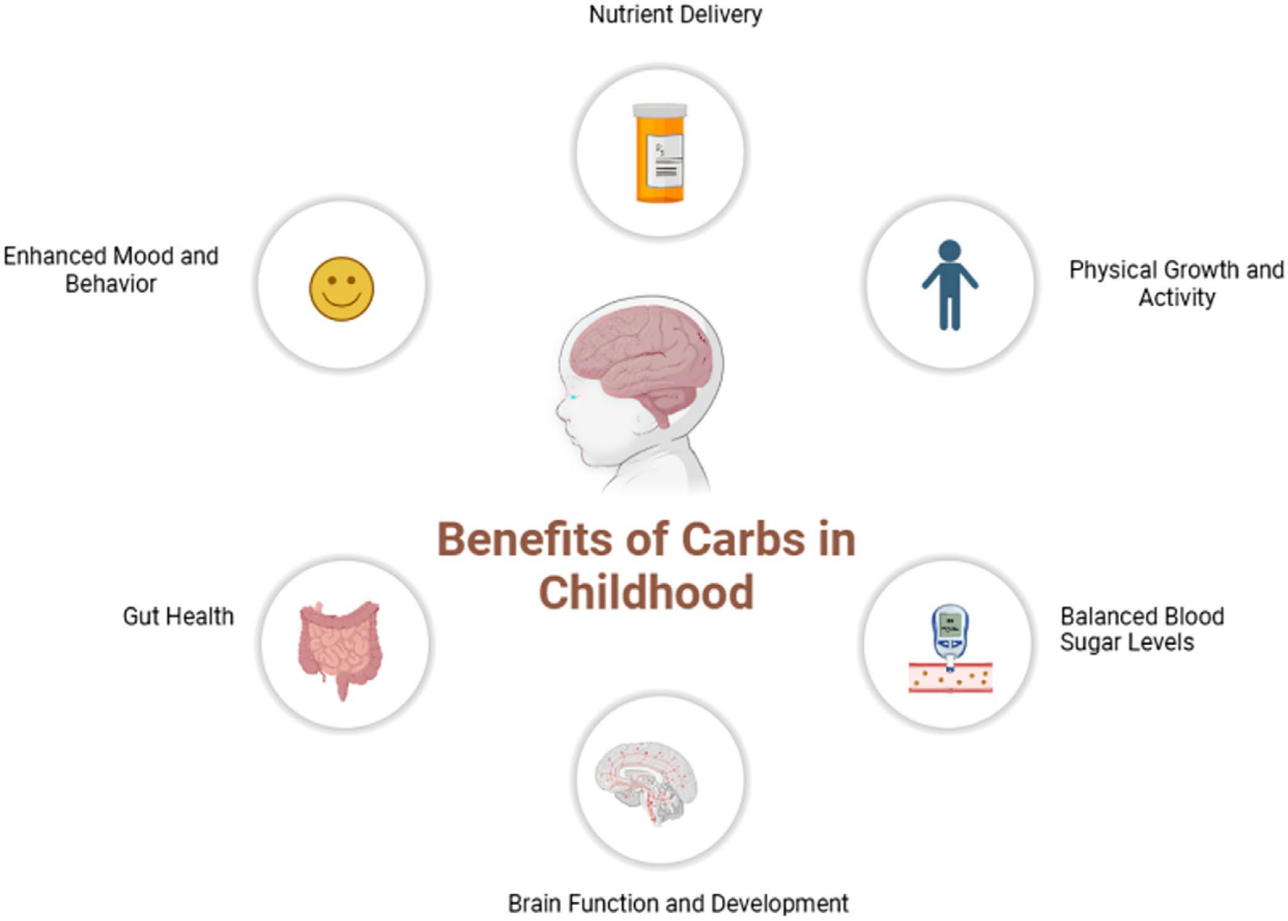
Benefits of carbs intake in childhood.

Cognitive impairments later in life have been linked to the effects of early‐life malnutrition on brain development in human populations (Langley‐Evans 2015). Hyperactivity, attention problems, and even neurodevelopmental disorders have been associated with diets in youth that are high in processed carbs and refined sugars (Kim et al. 2024). Children should be given a balanced carbohydrate diet to maintain long‐term cognitive health and immediate brain function (Figure 6).

### 7.2 Carbohydrates and Brain Function in Adulthood

Although the metabolic processes become more complicated, the brain still needs carbohydrates to function at the highest capacity as individuals enter their adult lives (Dai et al. 2023). The way that the brain metabolizes glucose largely depends on activity levels, insulin sensitivity, and metabolic rate among adults (Oleson et al. 2017). There is new evidence to propose that the brain will use substitute metabolites like lactate and ketones under conditions of low‐carbohydrate intake despite the fact that glucose remains the predominant source of fuel for the brain (Schurr 2014).

Diet and physical activity are two aspects of lifestyle that affect the relationship between carbohydrates and healthy adult brain function. For example, as González‐Reyes et al. (2016) describe, there has been a greater risk of obesity, metabolic syndrome, and type 2 diabetes associated with decreased brain function; all are more probable in individuals whose diets consist of high amounts of carbs and little fiber. Insulin resistance, one of the symptoms of such disorders, hampers glucose uptake by neurons and, subsequently, cognition (Goyal et al. 2017).

Conversely, studies indicate that a diet high in complex carbohydrates such as fruits, vegetables, and whole grains can improve brain health and insulin sensitivity and result in enhanced cognitive function (Kim et al. 2024).

Physical exercise plays a significant role in the glucose metabolism of the brain. Studies indicate that exercise increases glucose uptake by the brain, which can in turn improve cognition, memory, and learning (Meeusen 2014). In addition to that, Bustamante‐Sanchez et al. (2022) identified that exercise enhances brain‐derived neurotrophic factor protein synthesis, thereby enhancing synaptic plasticity, neurogenesis, and cognitive aging.

### 7.3 Carbohydrates and Brain Aging

An individual's brain's ability to uptake carbs effectively can decrease with age because of changes in anatomy and physiology. One of the key alterations that occur even in healthy aging is decreased brain glucose metabolism, according to Goyal et al. (2017). It is suspected that neurologic diseases such as Alzheimer's and age‐related cognitive impairment are rendered more probable by such a reduction in glucose use. Impaired glucose metabolism within the brain is one of the earliest signs of Alzheimer's pathology.

Goyal et al. (2017) suggest that this metabolic dysfunction can occur years prior to the onset of clinical symptoms. Consumption of carbs in controlled level as we develop older can contrary these variations, proposes the investigation. Le Couteur et al. (2016) exposed that a diet ironic in fruits, vegetables, and whole grains and squat in refined sugars might improve brain functions by improving glucose metabolism and inflammation decrease.

In accumulation, there have been numerous investigations that demonstrate that a decrease in glucose with an upsurge in ketones may be more operative in brain fuel metabolism and will advance cognitive functioning in elder age through carb restriction and the beginning of low‐carb or ketogenic diets (Zhu et al. 2015).

More investigation is required to govern the possessions of such dietary habits on brain role as well as on cognitive worsening. The association among carbs and brain aging is also prejudiced by the gut microbiome. Carbohydrates in the diet can modify the conformation of the gut microbiota, which consequently affects the brain through the gut–brain axis, based on existing investigations (Schneider et al. 2024).

Allen et al. (2017) articulate that, for instance, dietary consumptions greater in fiber comprising carbohydrates endorse the proliferation of advantageous gut bacteria. These bacteria then yield short‐chain fatty acids, which own neuroprotective properties and are maybe proficient at constraining inflammation in the brain. Thus, whether simple or complex, the carbohydrates consumed during aging could significantly impact metabolic and cognitive health.

### 7.4 Malnutrition and Its Effects on Cognitive Function

Malnutrition at any age, especially in carbohydrate insufficiency, can be devastating. Malnutrition during childhood may alter brain development, leading to behavioral issues and intellectual impairments that persist into adulthood (Vaiserman 2014).

There is a link between inadequate carbohydrate intake and poor cognitive performance in early life, especially among low‐income populations with limited access to nutrient‐dense meals (Rong et al. 2018). Since the brain is most vulnerable to dietary deficiencies at critical periods of growth and development, early‐life malnutrition has a particularly ominous impact on cognitive function (Alamy and Bengelloun 2012).

Poor eating habits can increase the risk of metabolic diseases such as obesity, diabetes, and cardiovascular disease in adulthood, especially if they are rich in refined carbs (Huang et al. 2024). Since these disorders directly affect glucose metabolism and promote inflammation, they directly impact brain health and are associated with neurodegeneration and cognitive decline (González‐Reyes et al. 2016).

In addition, because high sugar levels in the blood can lead to the formation of harmful amyloid plaques in the brain, a long time of high sugar consumption has been associated with a heightened risk of Alzheimer's disease (Kubis‐Kubiak et al. 2020).

Old age is associated with malnutrition and could increase the risk of patients who have dementia in conjunction with worsening age‐related cognitive deterioration. A deficiency in a vital nutrient, such as carbs, lowers neuronal energy supply and compromises neuroplasticity, leading to faulty brain function (Goyal et al. 2017).

Low‐carbohydrate consumption in older adults may imply less glucose available in the brain, which can be worse for aging‐related cognitive deficiencies (Schurr 2014). During life, carbohydrates affect the working of the brain and cognitive fitness. Proper consumption of carbohydrates throughout adulthood fosters cognitive ability and good metabolic health. Enough carbohydrate supply at the age of childhood ensures maximum possible brain growth. Preserving the equilibrium level of glucose metabolism with aging plays a pivotal role in avoiding neurodegenerative diseases and preserving cognitive capacity. The strong impact malnutrition at any life stage can have on brain health—whether caused by inadequate carbohydrate consumption or poor eating habits—highlights the need for proper nutrition at every stage of life. Further research will need to continue to study these complex interactions among carbohydrates, brain metabolism, and cognitive function to support dietary recommendations and therapeutic interventions focused on optimizing brain health across the life cycle.

### 7.5 Carbohydrates for Cognitive Development and Learning

As the brain rapidly grows, the brain relies on carbohydrates for energy in childhood. The brain requires constant glucose infusion, mainly from carbs, to maintain optimal cognitive functioning. The human brain undergoes significant development and neurogenesis between birth and adolescence, making the period especially sensitive to nutrient effects (Stephen et al. 2012).

Some cognitive processes supported by adequate carbohydrate intake include learning, memory, attention, and executive function. The growth of neurotransmitter systems necessary for cognition, synaptic plasticity, and the creation of new neural connections also depends on glucose availability (Alamy and Bengelloun 2012).

According to several research findings, poor carbohydrate intake during childhood is associated with reduced cognitive development. Undernourished children particularly those who came from low‐class families are prone to delayed motor skill development, lower IQ, and slower cognitive growth (Langley‐Evans 2015).

Insufficient glucose supply throughout critical eras can also influence brain functioning, which would make it more problematic for neuronal circuits related to learning to grow. An added prospective effect of insufficient carb consumption is heightened behavioral matters, including learning weakening and trouble concentrating (Vaiserman 2014). Henceforth, it is vital to make sure children take the right quantity of carbs for their brain growth and performance at school. Numerous carbohydrates produce varying effects on cognitive functioning. A diet high in complex carbohydrates, like those found in fruits, vegetables, and whole grains, has been found to supply the brain with stable glucose, which improves its capability to function systematically and efficiently (Stephen et al. 2012).

As specified by Sünram‐Lea and Owen (2017), when persons eat a diet ironic in simple carbohydrates and refined sugar, their blood sugar levels vary, thereby persuading their mood, reasoning, and capability for concentration and consideration. In accumulation, the quantity and varieties of carbohydrates taken beginning at infancy frequently shape subsequent cognitive capability development.

Additionally, investigation has revealed that the consumption of carbohydrates throughout early infancy can have long‐lasting influences on brain growth. For illustration, investigations have established that children who eat diets loaded with treated foods and sweet beverages accomplish inferior cognitive role and at school compared to children who consume diets that are greater in complex carbohydrates (Stephen et al. 2012).

Besides, examinations have demonstrated that the gut microbiota, which is related to cognitive role and mind health, is aided by carbohydrate consumption from good fiber sources (Allen et al. 2017). Thus, ensuring that the child consumes adequate amounts of good‐quality carbohydrates is essential for promoting academic performance and enhancing cognitive development.

### 7.6 Carbohydrate's Role in Mitigating Cognitive Decline in Older Adults

Anatomical and functional variations in the mind due to old age can subsidize to cognitive weakening. A significant metabolic change that occurs with age is a reduction of glucose utilization in brain cells, which is the major source of energy for brain cells (Goyal et al. 2017).

Glucose metabolism in the elderly decreases with age and is associated with neurological disorders such as Alzheimer's. Having a proper supply of glucose to the brain is essential to avoid or delay cognitive impairment in older people. Researchers argue that changes in diet can reduce the impact of aging on cognitive function through the facilitation of stable glucose metabolism.

Carbohydrates found in fruits, vegetables, and whole grain foods (Oleson et al. 2017) sustain memory and cognitive function by supplying the brain with constant glucose. Dementia and cognitive decline are more likely to result in individuals with metabolic dysfunction and insulin resistance, both increased by the delayed digestion of complex carbohydrates over simple sugars (Le Couteur et al. (2016)).

On the other hand, a diet rich in added sugars and refined carbohydrates has been linked to a higher cognitive decline risk (Kubis‐Kubiak et al. 2020) because such foods have the potential to worsen insulin resistance, inflammation, and oxidative stress in the brain. There is also evidence that carbohydrate restriction specifically, a low‐carb or ketogenic diet, may preserve neural function in older individuals.

Some studies have shown that changing the brain's primary fuel from glucose to ketones enhances cognitive function, particularly in individuals with early‐stage Alzheimer's disease (Zhu et al. 2015). When glucose metabolism is impaired, ketones, produced during periods of decreased carbohydrate intake, provide neurons with an alternative energy source and contribute to the preservation of brain function (Zhu et al. 2015).

Preliminary research suggests that ketogenic diets may alleviate some of the cognitive symptoms associated with neurodegenerative diseases. However, the long‐term effects of these diets on aging brains are still under investigation (Basiri et al. 2023).

Beyond the direct impact of carbohydrates on brain metabolism, a new study even proposed that gut microbiota participates in cognitive decline with age. Like fruits and vegetables, fiber‐rich carbohydrates promote beneficial gut flora growth, producing neuroprotective short‐chain fatty acids (Schneider et al. 2024). These SCFAs could promote healthy neuronal function, reduce inflammation within the brain, and enhance cognitive function. A diet high in fiber and complex carbs may thus help defend the brain against age‐related cognitive decline by supporting a healthy gut–brain axis.

Carbohydrates also influence mood and mental health, which are necessary for maintaining cognitive function in older adults. It has been shown that depression and chronic stress, which are more common in older populations, impact cognitive performance.

According to Foula and Foad (2024), carbohydrates, mainly those in high tryptophan‐containing foods, such as fruits and whole grains, enhance the secretion of serotonin, which increases mood and potentially reduces the likelihood of cognitive impairment. Carbohydrate consumption is also associated with better sleep, essential for cognitive processing and memory retention (Sünram‐Lea and Owen 2017).

Ensuring older adults consume adequate amounts of carbohydrates, primarily from good sources, may enhance their mood and cognitive functioning. Therefore, carbohydrates play a vital role in children's cognitive development and prevention of age‐related cognitive decline in older adults. Carbohydrates play an important role in children's brain development, learning, and cognitive functions, whereas complex carbohydrates are especially beneficial for long‐term health (Kuban et al. 2024).

While dietary patterns that emphasize whole, fiber‐rich carbs might protect the brain from neurodegenerative illnesses, proper carbohydrate intake is necessary to maintain a constant glucose supply and prevent cognitive loss in older persons. Thus, dietary treatments emphasizing the quantity and quality of carbohydrates may significantly impact cognitive health throughout life.

## 8 Carbohydrates, Gut–Brain Axis, and Mental Health

The investigation on the link among gut flora, brain role, and dietary carbs is just in the initial stage, but its demonstrations how significant food is for psychological health. Carbohydrates, particularly fermentable ones with greater fiber, directly impact the gut microbiota conformation. Baking soda, propionate, and acetate are short‐chain fatty acids formed when microorganisms in the gut digest these carbohydrates.

Allen et al. (2017) and Schneider et al. (2024) concluded that these SCFAs have neuroprotective possessions and disturb brain functioning. SCFAs can hypothetically subsidize to neurogenesis, decrease gut and brain inflammation, and accomplish temper and cognition, as per studied by Schneider et al. (2024). For a strong gut–brain axis, which accomplishes performance, feelings, and cognitions (Horovitz 2024), there requirements to be a various gut flora. A greater consumption of fermentable fibers grows these bacteria.

Critical mood, cognitive procedures and neurotransmitters the gut microbiota yield metabolites that effect these systems (Allen et al. 2017). Carbohydrates, particularly complex carbs present in fruits, vegetables, and whole grains, uphold the stomach's microbiome in balance by provided that helpful bacterium with prebiotics, as specified by Mate et al. (2021). By contrast, López‐Taboada et al. (2020) stated that mental health illnesses, inflammation, raised intestinal permeability, and dangerous bacterial development can be reason by high‐refined carb and simple sugar diets. Kaplan et al. (2015) exposed that this shift is related to mental illnesses such as anxiety, unhappiness, and damaged cognition, implying that nutrition plays a vital role in psychological health by changing the gut–brain association.

Prebiotics and fermentable fibers, which are nondigestible carbohydrates that human enzymes are incapable to break down but which feedstuff vigorous bacteria in the stomach, have been found to have an extensive effect on psychological health (Chen et al. 2025). These fibers improve the numbers of healthy gut bacteria, thereby improving the output of brain healthy metabolites such as SCFAs, conferring to Schneider et al. (2024).

To avert neurodegenerative illnesses and psychiatric disorders such as anxiety and depression, having substances such as butyrate, an SCFA, which owns anti‐inflammatory and oxidative stress decreasing possessions in the brain is significant (Allen et al. 2017). Furthermore, prebiotics avert the deprivation of the blood brain barrier, thereby averting damaging substances from attainment into the brain and can affect how we regulate our feelings and perceive (Horovitz 2024).

The fabrication of neurotransmitters by gut flora also be contingent on fiber and prebiotics. One of the neurotransmitters that manufactured by the gut flora in huge quantities is serotonin, which is accountable for temperament and behavior regulation. Investigation designates that a diet ironic in prebiotics is capable to improve mental health through the improvement of serotonin levels as well as endorsing the growth of positive gut flora (Chianese et al. 2018). In accumulation, as specified by Marano et al. (2023), mental problems can be treated or evaded by impacting the gut microbiota by exhausting dietary treatments such as prebiotics and high‐fiber diets.

Bananas, oats, and other foods good in fiber comprise prebiotics, which have been exposed in animal and human investigation to suggestively improve mood and decrease symptoms of nervousness and depression (Schneider et al. 2024). For instance, investigations have related fermentable fiber‐dense diets with abridged nervousness and stress, perhaps due to an enhanced balance of neurotransmitters and abridged inflammation in the gut (Kaplan et al. 2015). Patients with temperament disorders, unhappiness, or cognitive damage can advantage by comprising fermentable fibers and prebiotics in their diets.

## 9 Practical Implications and Dietary Recommendations

The normal brain function and mental well‐being are reliant on achieving the correct proportion of carbohydrates every day. Research has revealed that a high‐carbohydrate diet can produce beneficial impacts on cognitive function, emotional control, and general mental well‐being. To achieve balance in blood sugar and avert loss of cognitive function, healthy grains and vegetables should be consumed in moderation. Such foods contain low glycemic carbohydrates (Tzenios and Wong 2024).

On the other hand, fast digesting, high glycemic carbs cause emotional instability and impair cognitive function (Shahpasand et al. 2024). Moreover, because they furnish an alternative source of energy, ketones, the ketogenic diet, which significantly reduces carbohydrates, has been shown to improve mood and cognitive efficiency, especially in individuals with psychiatric or neurodegenerative disease (Anderson et al. 2024). These studies underscore the importance of carb intake with consciousness for mental and emotional health (Garner et al. 2024).

Personalized diet is increasingly recognized for enhancing mental and psychological well‐being. Different nutritional methods may be needed for each person depending on their metabolic profile, genetic predisposition, and psychological state. A ketogenic or low‐carb diet, for instance, might be useful to those with mood disorders or neurodegenerative diseases because it has been proven to improve emotional stability and cognitive function (Mentzelou et al. 2023).

Other people might benefit more from the Mediterranean‐style diet, promoting brain function and reducing inflammation, through consuming modest amounts of fruits, whole grains, and healthy fats (Field et al. 2023). Artificial intelligence is a promising new area for enhancing mental health outcomes, which can be used to develop tailored food plans based on psychological needs and metabolic responses (Tzenios and Wong 2024). Therefore, tailored nutrition offers a tailored approach that enhances the potential benefits of nutritional interventions on outcomes concerning mental and physical health (Shahpasand et al. 2024).

In summary, the customization and fine‐tuned approach of nutrition to carbohydrates is the requirement for optimally maximized brain health and psychological well‐being. Personalized dietary recommendations address the prevention and management of several mental illness conditions, considering specific metabolism and psychological characteristics. Therefore, it promises preventive and treatment methods (Garner et al. 2024; Mentzelou et al. 2023).

## 10 Conclusion and Future Perspectives

The value of carbs for psychological wellness and optimal mental performance is accentuated here. Glucose and other carbs play a marvelous role in manipulating the brain's cognitive, emotional, and psychological functioning. This emphasizes the opposite actions of simple and complex carbs, with low‐glycemic index carbs that augment steady psychological clarity and endurable focus and high‐glycemic index carbs related to mood fluctuation and lower mental effectiveness.

The fabrication of neurotransmitters, such as the mood‐modulating neurotransmitter serotonin, is an additional mechanism by which carbohydrates endorse mental health. However, there are psychosocial and physiological hazards to very low‐carbohydrate and ketogenic diets that require close monitoring in order to assist from the possible benefits of improving cognitive function, particularly in neurological disorders. Carbohydrate consumption and brain functioning also diverge across life, manipulating the cognitive development of children and the capability of adults to resist aging's impact on their brain power. Recent investigation on the gut–brain axis designates that prebiotics and fermentable fibers could have an influence on psychological health and that personalized diet plans can improve psychological health consequences. Generally, these findings reinforce the requirement for further investigation to fine‐tune evidence‐based direction, and these again affirm the significance of personalized dietary strategies in endorsing mental health.

Future research in nutrition psychology should focus on studying the gut–brain axis and how it affects how carbs impact brain functioning. The studies regarding the effect of fermentable fibers, prebiotics, and microbiotas on mental health are highly promising. Additionally, customized nutrition approaches are needed according to the individual's metabolic profile and psychological conditions to get the best possible brain health and avoid mental health diseases. Expanding our knowledge of carbohydrates' biochemical, physiological, and psychological effects on brain function may help us design even more tailored, efficient diets that work throughout life to enhance cognitive functioning and emotional well‐being.

## Author Contributions

**Muhammad Tayyab Arshad:** methodology (equal), writing – original draft (equal). **Sammra Maqsood:** data curation (equal), writing – review and editing (equal). **Rawan Altalhi:** conceptualization (equal), data curation (equal). **Ghalia Shamlan:** data curation (equal), formal analysis (equal). **Isam A. Mohamed Ahmed:** data curation (equal), formal analysis (equal). **Ali Ikram:** supervision (equal), validation (equal). **Muhammed Adem Abdullahi:** project administration (equal), writing – original draft (equal).

## Disclosure

The authors have nothing to report.

## Ethics Statement

This study did not involve humans or animals.

## Consent

This study did not involve humans.

## Conflicts of Interest

The authors declare no conflicts of interest.

## Data Availability

The data that support the findings of this study are available upon request from the corresponding author. The data are not publicly available due to privacy or ethical restrictions.
